# Effects of resveratrol on growth performance, meat quality, and intramuscular fat deposition in finishing steers

**DOI:** 10.1093/jas/skaf410

**Published:** 2025-11-25

**Authors:** Ming Yang, Kaixi Ji, Xiaomu Liu, Zhonghua Wang, Xueyan Lin, Hongbo Zhao, Xianglun Zhang

**Affiliations:** Institute of Animal Science and Veterinary Medicine/Shandong Academy of Agricultural Sciences, Key Laboratory of Livestock and Poultry Multi-omics of MARA, Shandong Key Laboratory of Animal Microecologics and Efficient Breeding of Livestock and Poultry, Jinan 250100, China; College of Animal Science and Technology, Shandong Agricultural University, Taian 271000, China; Institute of Animal Science and Veterinary Medicine/Shandong Academy of Agricultural Sciences, Key Laboratory of Livestock and Poultry Multi-omics of MARA, Shandong Key Laboratory of Animal Microecologics and Efficient Breeding of Livestock and Poultry, Jinan 250100, China; Institute of Animal Science and Veterinary Medicine/Shandong Academy of Agricultural Sciences, Key Laboratory of Livestock and Poultry Multi-omics of MARA, Shandong Key Laboratory of Animal Microecologics and Efficient Breeding of Livestock and Poultry, Jinan 250100, China; College of Animal Science and Technology, Shandong Agricultural University, Taian 271000, China; College of Animal Science and Technology, Shandong Agricultural University, Taian 271000, China; Institute of Animal Science and Veterinary Medicine/Shandong Academy of Agricultural Sciences, Key Laboratory of Livestock and Poultry Multi-omics of MARA, Shandong Key Laboratory of Animal Microecologics and Efficient Breeding of Livestock and Poultry, Jinan 250100, China; Institute of Animal Science and Veterinary Medicine/Shandong Academy of Agricultural Sciences, Key Laboratory of Livestock and Poultry Multi-omics of MARA, Shandong Key Laboratory of Animal Microecologics and Efficient Breeding of Livestock and Poultry, Jinan 250100, China

**Keywords:** intramuscular fat, meat quality, PPAR signaling pathway, resveratrol, steer

## Abstract

This study examined the impact of resveratrol (RES) on growth performance, meat quality, and intramuscular fat (IMF) deposition in steers undergoing finishing. Thirty healthy Angus steers with similar body weights (mean 571.83 ± 5.27 kg) were divided into three groups (*n* = 10) and fed the following diets for 240 d: 1) basal diet, 2) basal diet containing 400 mg/kg RES, or 3) basal diet containing 800 mg/kg RES. The results showed that supplementation with 400 mg/kg RES significantly increased the final body weight and average daily gain of steers (*P *< 0.05), whereas the 800 mg/kg group did not show a significant effect. Moreover, 400 mg/kg RES significantly increased the live weight before slaughter and carcass weight while reducing subcutaneous backfat thickness (*P *< 0.05). Additionally, both levels of RES supplementation significantly increased the IMF content in the *Longissimus lumborum* (LL) muscle and reduced drip loss, cooking loss, and shear force (*P *< 0.05). Notably, 400 mg/kg RES supplementation significantly decreased adipose triglyceride lipase and hormone-sensitive lipase activities of LL muscle and acetyl-CoA carboxylase activity was enhanced in all RES-treated groups (*P *< 0.05). These results integrating transcriptomics, proteomics, real-time quantitative PCR, and Western blotting revealed that RES upregulated both the mRNA and protein expression levels of fatty acid transport-related factors (fatty acid transport protein 1, fatty acid-binding protein 3, and fatty acid-binding protein 4) and lipogenesis-related factors (acetyl-CoA carboxylase and diacylglycerol O-acyltransferase 1) in the PPAR signaling pathway of the LL muscle. Collectively, dietary 400 mg/kg RES improved growth performance and carcass traits, specifically enhancing meat quality and promoting IMF deposition of the LL muscle. These effects were primarily mediated through activating the peroxisome proliferator-activated receptor signaling pathway, which enhances the expression of factors related to fatty acid uptake and transport, lipid synthesis.

## Teaser

Supplementing cattle feed with resveratrol, particularly at 400 mg/kg, improves growth performance and meat quality while especially increasing the intramuscular fat content of *Longissimus lumborum* muscle by modulating fat metabolism through the PPAR signaling pathway.

## Graphical Abstract

**Graphical Abstract:**
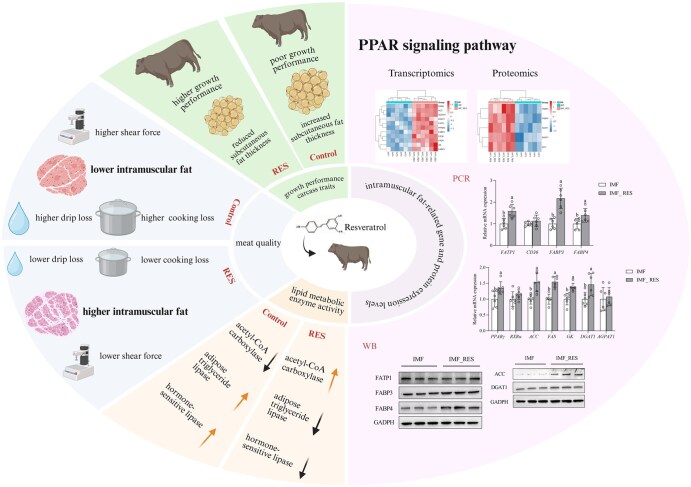

## Introduction

As a typical representative of high-end raw meat, marbled beef is highly favored by consumers due to its tenderness, juiciness, and unique aroma (Chang et al., 2022). The marbling score is a key indicator for assessing intramuscular fat (IMF) content; a higher marbling score indicates greater IMF content and better beef grade (Schumacher et al., 2022). However, IMF deposition in beef cattle is associated with prolonged feeding cycles and low feeding efficiency (Park et al., 2018). Therefore, improving IMF deposition efficiency and reducing feeding costs through nutritional regulation technologies have become key research focuses in this field.

Natural plant extracts have attracted significant attention for their efficacy in regulating lipid metabolism in livestock (Álvarez-Rodríguez et al., 2022). Resveratrol (RES), a natural polyphenolic plant extract, has been extensively studied for its role in regulating lipid metabolism (Cheng et al., 2020), enhancing the antioxidant capacity of livestock (Meng et al., 2018), improving immune function (He et al., 2019), and promoting intestinal health (Yang et al., 2021).

Some studies have shown that RES affects IMF deposition in livestock and poultry, but the findings across these studies are inconsistent (Zhang et al., 2015, 2019; Jin et al., 2021; Shen et al., 2022). On the one hand, RES can promote IMF deposition. For example, Zhang et al. (2019) revealed that dietary RES supplementation significantly promoted IMF deposition in the *longissimus dorsi* (LD) of finishing pigs, and this effect is related to the sirtuin 1-peroxisome proliferator-activated receptor gamma (SIRT1-PPARγ) signaling pathway. Shen et al. (2022) reported that adding RES to the diet of goats improved IMF content, although the specific mechanism remains unclear. On the contrary, some studies reported that RES had no effect on or inhibited IMF deposition. Zhang et al. (2015) observed that IMF deposition in the LD muscle was not affected by RES supplementation in finishing pigs. Jin et al. (2021) found that the IMF content in the breast muscle of ducks decreased linearly with increasing RES addition. Therefore, the reported effects of RES on IMF deposition in livestock and poultry are controversial, and the underlying molecular mechanisms remain unclear. Moreover, no relevant studies have been conducted on beef cattle production. To fill this gap in the knowledge, we carried out this research on the application of RES in finishing cattle. We hypothesized that dietary RES supplementation would improve growth performance, enhance meat quality, and promote IMF deposition in finishing steers.

## Materials and Methods

### Animals, experimental design, and management

The animal experimentation protocol was ethically reviewed and authorized through the institutional oversight mechanism at Shandong Academy of Agricultural Sciences (SAAS-2024-067). Thirty healthy Angus steers (mean body weight of 571.83 ± 5.27 kg) raised on a commercial farm in Dezhou City, Shandong Province, China (36°24'N, 116°23'E) were selected for the trial. Prior to the experiment, all steers underwent a 15-d adaptation period. During this period, all steers were housed in individual pens and fed the uniform basal diet. The steers were then randomly allocated into three dietary treatment groups, with ten replicates in each group: a control group (basal diet) and two treatment groups, supplemented with either 400 or 800 mg/kg RES.

The basal diet was designed in compliance with NRC (2016) guidelines to fulfill the nutrient requirements of beef cattle. The composition and nutrient content of the basal diets are shown in Table 1. Resveratrol was supplied by XI’AN Feida Bio-Tech Co., Ltd (Xi’an, China), with a 98.1% purity. The feeding experiment lasted 240 d. Throughout the experimental period, steers were housed in individual pens and fed twice daily at 07:00 and 17:00, with *ad libitum* access to potable water. Final body weights were recorded at the termination of the experiment, and average daily gain was derived from the difference between initial and final body weight. Daily feed provision and residual quantities were systematically recorded to calculate dry matter daily intake.

**Table 1.** Composition and nutrient levels of diet (dry matter basis)

Ingredient^1^	Content (%)	Nutrient level	Content (%)
**Straw**	20.00	Net energy for gain (MJ/kg)	5.41
**Corn flakes**	55.00	Crude protein	12.67
**Soybean meal**	11.00	Ether extract	2.02
**Wheat bran**	10.00	Neutral detergent fiber	27.66
**Limestone**	0.80	Acid detergent fiber	13.04
**NaCl**	0.80	Calcium	0.40
**NaHCO_3_**	1.60	Phosphorus	0.36
**Premix**	0.80		
**Total**	100.00		

1 The premix provided the following per kg of diets: 2,200 IU vitamin A, 1,500 IU vitamin D, 50 IU vitamin E, Cu 30 mg, Mn 35 mg, Fe 50 mg, Zn 120 mg, Se 7.5 mg, I 1.2 mg, Co 0.5 mg. Net energy for gain was derived through predictive equations based on nutrient composition, whereas all other nutrient parameters were determined via laboratory analyses

### Carcass measurements and sample collection

At the end of the experimental period, six steers per group were randomly selected and transferred to the abattoir (5 kilometers close from the farm) for slaughter. Following a 24-h fasting period, the animals were weighed before slaughter. Thereafter, the steers were exsanguinated via the aorta. After skinning and removing the head, hooves, tail, and visceral organs, the carcass weight was measured, and the dressing percentage was calculated. After skinning, IMF samples (approximately 10 g) were immediately collected from the left half-carcass between the 12th and 13th ribs using a circular drilled meat extractor (Yi et al., 2024). The samples were then rinsed with phosphate-buffered saline (PBS), snap-frozen in liquid nitrogen, and stored at –80 °C for subsequent analysis of lipid metabolism enzyme activities, proteomic, transcriptomic, real-time quantitative PCR (RT-qPCR), and Western blotting. The left side of the carcass was then chilled at 4 °C for 72 h (Boles et al., 2005). After chilling, the carcass was ribbed between the 12th and 13th ribs (Bohrer et al., 2014). The subcutaneous backfat thickness at the 12th to 13th rib interface as measured at a point 3 cm from the midline using vernier calipers (Li et al., 2022). The eye muscle area was determined by outlining the cross-sectional contour of the *Longissimus lumborum* (LL) muscle at the same location using transparent grid-ruled tracing paper (Bodwell et al., 1959). Meat color and pH were measured on the exposed LL muscle surface. Finally, approximately 1,000 g of the LL muscle was carefully dissected, ensuring the exclusion of accessory muscles and subcutaneous fat (Luan et al., 2023), and used for the measurement of meat quality and nutritional composition.

### Meat quality and nutritional composition analysis

Muscle pH values and color parameters (L* lightness; a* redness; b* yellowness) were measured at 72 h postmortem. The pH meter was calibrated prior to each measurement series using pH 4.0 and 7.0 standard solutions under controlled conditions at 25 °C. Meat color was assessed by a colorimeter (NR20XE; 3nh Technology, Shenzhen, China), calibrated before use using the supplied white and black tiles. The instrument was configured using a D65 light source, a 10° standard observer, and a 20-mm aperture (the measurement was conducted on a freshly cut surface after blooming for 30 min at 4 °C) (Bai et al., 2023). Both pH and color values were recorded in triplicate and averaged.

To determine drip loss, muscle samples were trimmed into 2 × 2 × 4 cm strips (W1) along the myofiber orientation, vertically suspended in polyethylene bags, and stored at 4 °C. They were reweighed (W2) after 24 and 48 h of suspension. Drip loss was calculated as: Drip loss (%) = [(W1 – W2)/W1] × 100% (He et al., 2023).

To determine cooking loss, meat samples measuring approximately 4 × 5 × 2 cm were weighed (W3) (Jin et al., 2021). All samples were cooked simultaneously in a single batch. Each sample was individually sealed in a heat-resistant vacuum bag to maintain its identity and integrity (He et al., 2023). All bags were fully submerged in a water bath maintained at 80 °C. The core temperature of every sample was monitored in real-time using a digital thermometer (905-T1, Testo SE & Co. KGaA, Titisee-Neustadt, Germany), with the probe inserted into the center of the meat. Heating was continued until the core temperature reached 70 °C (Yi et al., 2024). Subsequently, the samples were removed, cooled to ambient temperature, blotted dry on the surface, and reweighed (W4). Cooking loss was calculated as: cooking loss (%) = [(W3 – W4)/W3] × 100% (Li et al., 2025).

For the shear force analysis, the samples were vacuum-packed and heated in an 80 °C water bath until they reached an internal temperature of 70 °C, followed by cooling to room temperature and storage at 2 ± 2 °C overnight (Li et al., 2022). From each sample, eight cylindrical cores (11 mm in diameter) were drilled parallel to the myofiber orientation using a coring device. The shear force was then determined using a texture analyzer (TA. XT Plus C; Stable Micro Systems, Surrey, UK) fitted with an HDP/WBV 60° angular probe (Song et al., 2020). The instrument settings were as follows: pre-test speed of 2.0 mm/s, test speed of 1.0 mm/s, post-test speed of 5.0 mm/s, and trigger force of 5 g. Each core was positioned so that the blade sheared perpendicularly to the myofiber orientation as 23.0 mm. The peak force (N) required to shear the core was recorded as the shear force value (Li et al., 2022).

Approximately 300 g of LL muscle was weighed and freeze-dried using a lyophilizer (SCIENTZ-18N; Ningbo Scientz Biotechnology Co., Ltd, Ningbo, China). The freeze-dried samples were first pulverized, and their crude protein content was determined accurately according to the common standard of Kjeldahl nitrogen determination. Briefly, 0.5 g of pulverized sample was mixed with 8 mL of concentrated H_2_SO_4_ (98%), 1.2 g K_2_SO_4_, and 0.2 g CuSO_4_ and then digested in a digestion furnace until the solution was clarified. Thereafter, the digestate was transferred to a Kjeldahl nitrogen analyzer (Kjeltec 8400; FOSS, Hillerød, Denmark) to quantify the crude protein content on a dry matter basis (Benedict, 1987). The IMF content was measured using the Soxhlet extraction method. Briefly, freeze-dried and pulverized samples (0.5 g) were directly wrapped in filter paper. The filter paper package was placed in a Soxhlet extractor and extracted using anhydrous ether with a water bath temperature maintained at 45 °C for 6-7 h. After extraction, the samples were again oven-dried until a constant weight. The IMF content was calculated as the difference in weight before and after extraction as a percentage of the wet muscle tissue (Zhang et al., 2019).

### Lipid metabolic enzyme activity

A 2.5 g IMF sample was mixed with ice-cold physiological saline (4 °C) at a 1:9 mass-to-volume ratio. The mixture was homogenized for 15 s, followed by centrifugation at 4 °C and 12,000 × g for 10 min to isolate the supernatant. Enzyme-linked immunosorbent assay (ELISA) kits (Jiangsu Jingmei Biological Technology Co., Ltd) were employed to quantify the activities of lipid metabolic enzymes, including: fatty acid synthase (FAS; JM-08616B), acetyl-CoA carboxylase (ACC; JM-08593B1), stearoyl-CoA desaturase-1 (SCD1; JM-08596B1), adipose triglyceride lipase (ATGL; JM-08607B2), hormone-sensitive lipase (HSL; JM-08600B2), and carnitine palmitoyl transferase-1 (CPT-1; JM-08612B2). All assays were performed in strict accordance with the manufacturer’s protocols. The total protein concentration was determined using a bicinchoninic acid (BCA) assay kit (CW0014S; CWBio, Jiangsu, China).

### Transcriptomics and proteomics

#### Transcriptomic profiling and gene expression analysis

Approximately 100 mg of the IMF sample was collected for RNA extraction. Total RNA was then isolated via the TRIzol method. RNA integrity and quantity were evaluated with the Agilent 2100 Bioanalyzer (Agilent Technologies, Santa Clara, CA, USA). Qualified samples (1.5 μg RNA per group) were selected for sequencing. Enrichment of mRNA with oligo (dT) magnetic beads was followed by synthesis of double-stranded cDNA utilizing fragmented mRNA as a template. After end repair, A-tailing, and adapter ligation, cDNA libraries were constructed using PCR amplification. The library was checked for quality and sequenced using an Illumina NovaSeq 6000 platform (Illumina, San Diego, CA, USA). The sequencing data underwent filtration to eliminate low-quality reads and adapter sequences, obtaining clean reads. The HISAT2 software was employed to align sequences with the reference genome (version: *Ensembl_100_bos_taurus_ars_ucd1_2_toplevel*). Differentially expressed genes (DEGs) were identified using a significance criterion of *P*-value < 0.05 and |log_2_ (foldchange)| >1.

#### Proteomic profiling and protein abundance analysis

Following the method of Kachuk et al. (2015), the samples stored at −80°C were ground with liquid nitrogen. The resulting material was then subjected to ice-cold lysis through agitation in SDS-Dithiothreitol-Tris buffer containing 100 mM NaCl. Subsequently, the lysate was centrifuged at 4 °C (12,000 rpm) to isolate the supernatant fraction. The supernatant was treated with iodoacetamide and incubated in the absence of light for 1 h. The mixture was then precipitated with pre-cooled acetone at −20°C for 2 h. After washing and drying, the proteins were dissolved in Dissolved buffer. Protein concentration was measured via the Bradford assay, with compliant samples adjusted to a final volume of 100 μL. Thereafter, trypsin and CaCl_2_ were added, and the mixture was incubated at 37 °C overnight. After adjusting the pH to < 3, the filtrate was collected by centrifugation and lyophilized. The Liquid Chromatography-Tandem Mass Spectrometry (LC-MS/MS) analysis was conducted on a Thermo Orbitrap Astral mass spectrometer. The mobile phases employed were solution A (aqueous 0.1% formic acid) and solution B (0.1% formic acid in acetonitrile). The ion source was operated at 1.9 kV and maintained at 290 °C. Proteins were identified and quantified using DIA-NN software based on the Bos_taurus_2023_10_18. Fasta (47,128 sequences) protein database with a false discovery rate (FDR) < 1% (Demichev et al., 2020). Differentially abundant proteins (DAPs) defined as proteins with *P *< 0.05 and |log_2_ (foldchange)| > 0.263 (equivalent to fold change > 1.2, fold change < 0.83), were identified.

### RNA isolation, reverse transcription, and RT-qPCR

For RNA extraction, frozen tissues were homogenized in a liquid nitrogen pre-chilled mortar with continuous liquid nitrogen replenishment during grinding. The resulting powder was transferred to RNase-free 2-mL microcentrifuge tubes. Total RNA was isolated using an Ultrapure RNA Kit (CWBio Biotech) following the manufacturer’s protocol. The quantity and quality of RNA were confirmed via OD260/OD280 ratio measurements utilizing the ThermoFisher Technologies NanoDrop2000 instrument. First-strand cDNA was synthesized with HIScript II Q RT Super Mix (+gDNA wiper) (Vazyme, Nanjing, China). Quantitative PCR was performed on a Light Cycler 480 II system (Roche, Basel, Switzerland) using ChamQ SYBR Color qPCR Master Mix (Vazyme). Relative gene expression was normalized to *β-actin* and calculated using the 2^−ΔΔCt^ method. The primers for quantitative polymerase chain reaction, designed and synthesized by Biosune (Shanghai, China), are detailed in Table 2.

**Table 2.** Primers used for qPCR

Gene^1^	Primer sequence (5′–3′)	Product size (bp)
***β-actin***	F: CACCGCAAATGCTTCTAGGC R: TGTCACCTTCACCGTTCCAG	186
***FATP1***	F: TGATGGATGAGCTGGGCTAC R: TTTGCCCTCTACTCCTGGCA	167
***CD36***	F: TTGGTGATGAGAAGGCGGA R: ACCAACACTGAGCAAGACG	92
***FABP3***	F: AGTTCGATGAGACCACAGC R: TCAACCATCTCCCGCACAA	124
***FABP4***	F: GTTTGAATGGGGGTGTGGT R: ACGATGCTCTTGACTTTCCT	124
***PPARγ***	F: CCTTCCAACTCCCTCATGGC R: CCGGAAGAAACCCTTGCATC	105
***RXRα***	F: TCGGTCATCAGTTCCCCCAT R: TTGGTGAAGGACGCCATGTT	200
***FAS***	F: GTGGGCTTGGTGAACTGTCT R: AGGACTTCGGGTCTGTCTCA	112
***ACC***	F: TCGACCTGCTGGAGGAGAA R: GTAAGGCCAGACCATCCTGG	105
***GK***	F: AAATCTCTCATAGCCCGAAA R: GACTGGTCTCCCAAACACC	70
***DGAT1***	F: TGTCACTCATCATCGGGCAG R: CTCACGGTTGAGCACGTAGT	71
***AGPAT1***	F: CGTTGTCTCCAACCACCAGA R: GCGACCTCAGACATGACACT	192

1 β-actin, beta-actin; FATP1, fatty acid transport protein 1; CD36, cluster of differentiation 36; FABP3, fatty acid-binding protein 3; FABP4, fatty acid-binding protein 4; PPARγ, peroxisome proliferator-activated receptor gamma; RXRα, retinoid X receptor alpha; FAS, fatty acid synthase; ACC, acetyl-CoA carboxylase; GK, glycerol kinase; DGAT1, diacylglycerol O-acyltransferase 1; AGPAT1, 1-acylglycerol-3-phosphate O-acyltransferase 1.

### Western blotting analysis

Total and nuclear proteins were extracted using radioimmunoprecipitation assay buffer (Beyotime, Shanghai, China) following the manufacturer’s protocol. Protein concentrations were determined using a BCA assay kit (P0010; Beyotime). Following electrophoresis of equal protein quantities via SDS-PAGE, the samples were transferred to polyvinylidene fluoride membranes (ISEQ00010; Millipore, Billerica, MA, USA). The membranes were then blocked using 5% skimmed milk for 1 h before being incubated overnight at 4 °C with the specified primary antibodies: anti-fatty acid transport protein 1 (FATP1) (CQA5932; Cohesion Biosciences Limited, Suzhou, China), anti-fatty acid-binding protein 3 (FABP3) (10676-1-AP; Proteintech, Wuhan, China), anti-FABP4 (12802-1-AP; Proteintech), anti-ACC (bs-11912R; Bioss Biotechnology, Beijing, China), anti-diacylglycerol O-acyltransferase 1 (DGAT1) (CQA3228; Cohesion Biosciences Limited), and anti-GAPDH (60004-1-lg; Proteintech). Following three washes with TBST buffer (containing 0.05 M Tris, 0.15 M NaCl, and 0.1% Tween-20), the blots were incubated with secondary goat anti-rabbit or goat anti-mouse secondary antibodies (A0216/A0208; Beyotime) at room temperature for 2 h. After three additional TBST washes, the protein bands were visualized using an enhanced chemiluminescence (ECL) substrate (180-5001; Tanon Science & Technology Co., Ltd, Shanghai, China) and detected using a chemiluminescence imaging system (Tanon 5200; Tanon Science & Technology Co., Ltd). Band intensity was quantified using Quantity One software (Bio-Rad Laboratories, Hercules, CA, USA).

### Statistical analysis

Following initial data collation, statistical analysis was performed using R programming language (v4.2.1; R Foundation for Statistical Computing, Vienna, Austria). One-way ANOVA was performed to assess the dietary treatments (fixed effect) on the differences in growth and carcass traits, meat quality, conventional nutritional components of the LL muscle, and lipid metabolic enzyme activities of finishing steers. All data are reported as the means with the standard error of the mean (SEM) in a separate column, and *P*-value of less than 0.05 denotes statistical significance. Gene and protein expression data were analyzed using an independent two-sample *t*-test and visualized as bar graphs using GraphPad Prism (v10.4.1; GraphPad Software, La Jolla, CA, USA).

Bioinformatics analysis was conducted using the R programming language. Transcriptomic data underwent differential gene expression analysis using the DESeq2 (v1.20.0) package, while proteomic data were subjected to differential protein abundance analysis employing Student’s *t*-test. Kyoto Encyclopedia of Genes and Genomes (KEGG) pathway enrichment was determined via the “clusterProfiler” package, and the results were visualized as bubble plots. Additional visualizations included volcano plots (generated using “ggplot2”) and heat maps (constructed using “pheatmap”).

## Results

### Effect of dietary RES on growth performance in finishing steers

As shown in Table 3, there were no differences (*P *> 0.05) in dry matter daily intake of finishing steers across all groups. Compared to the control group, the steers in the 400 mg/kg RES group exhibited a significant increase in final body weight and average daily gain (*P *< 0.05), whereas no significant effect was observed in the 800 mg/kg RES group (*P *> 0.05).

**Table 3.** Effects of dietary RES on growth performance in finishing steers

Items^1^	Treatments			SEM	*P*-value
	Control	400 mg/kg	800 mg/kg		
**Initial body weight, kg**	570.30	574.10	571.10	5.27	0.956
**Final body weight, kg**	726.50^b^	753.40^a^	735.20^a,b^	4.49	0.038
**Average daily gain, kg**	0.65^b^	0.75^a^	0.68^a,b^	0.01	0.016
**Dry matter daily intake, kg**	10.94	10.94	10.94	0.20	0.999

1 Abbreviations: Control, basal diet; 400 mg/kg, basal diet supplemented with 400 mg/kg resveratrol; 800 mg/kg, basal diet supplemented with 800 mg/kg resveratrol.

Thirty animals were used. Results are presented as mean, with SEM listed separately (*n* = 10 biological replicates). Distinct superscript letters within the same row denote statistically significant intergroup differences (*P *< 0.05).

### Effect of dietary RES on carcass traits in finishing steers

As shown in Table 4, varying RES supplementation levels demonstrated no impact (*P *> 0.05) on dressing percentage and eye muscle area across all experimental groups of finishing steers. Compared to the control group, the steers in the 400 mg/kg RES group exhibited a significant increase in live weight before slaughter and carcass weight (*P *< 0.05), while also showing a significant decrease in subcutaneous backfat thickness (*P *< 0.05). No significant effect on carcass traits was detected in the 800 mg/kg RES group (*P *> 0.05).

**Table 4.** Effects of dietary RES on carcass traits in finishing steers

Items^1^	Treatments			SEM	*P*-value
	Control	400 mg/kg	800 mg/kg		
**Live weight before slaughter, kg**	703.67^b^	731.17^a^	714.83^a,b^	4.47	0.029
**Carcass weight, kg**	454.97^b^	481.83^a^	467.80^a,b^	4.58	0.046
**Dressing percentage, %**	64.64	65.90	65.49	0.52	0.628
**Eye muscle area, cm^2^**	92.46	94.43	93.54	0.81	0.640
**Subcutaneous backfat thickness, cm**	2.39^a^	2.12^b^	2.26^a,b^	0.05	0.048

1 Abbreviations: Control, basal diet; 400 mg/kg, basal diet supplemented with 400 mg/kg resveratrol; 800 mg/kg, basal diet supplemented with 800 mg/kg resveratrol.

Eighteen animals were used. Results are presented as mean, with SEM listed separately (*n* = 6 biological replicates). Distinct superscript letters within the same row denote statistically significant intergroup differences (*P *< 0.05).

### Effect of dietary RES on meat quality and nutrient composition of LL muscle in finishing steers

**Figure 1.**
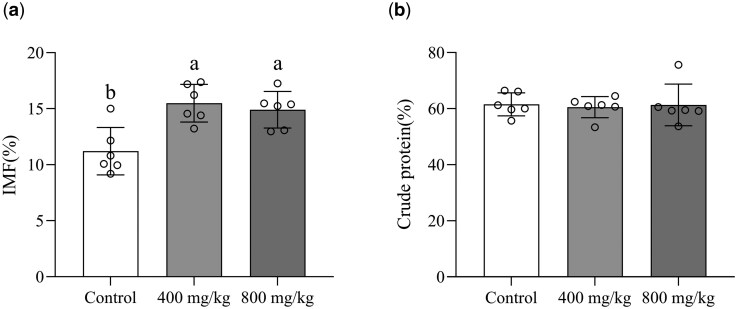
Effects of dietary resveratrol on intramuscular fat (a) and crude protein (b) in the *Longissimus lumborum* muscle of finishing steers. Different lowercase letters above the bars indicate statistically significant differences between groups (*P *< 0.05). Eighteen animals were used. Results are presented as mean, with SEM listed separately (*n* = 6 biological replicates). IMF, intramuscular fat; Control, basal diet; 400 mg/kg, basal diet supplemented with 400 mg/kg resveratrol; 800 mg/kg, basal diet supplemented with 800 mg/kg resveratrol.

As shown in Table 5, there were no differences (*P *> 0.05) in pH and meat color of the LL muscle among the groups. However, both the 400 and 800 mg/kg RES groups exhibited lower drip loss, cooking losses and shear force in the LL muscle compared to the control group (*P *< 0.05). While crude protein content showed no intergroup variation (Figure 1, *P *> 0.05), the IMF content in the 400 mg/kg RES (15.50%) and 800 mg/kg RES (14.90%) groups was increased compared to the control (11.20%, *P *< 0.05).

**Table 5.** Effects of dietary RES on meat quality of *longissimus lumborum* muscle in finishing steers

Items^1^	Treatments			SEM	*P*-value
	Control	400 mg/kg	800 mg/kg		
**pH_72 h_**	5.79	5.81	5.71	0.03	0.459
L*_72h_	43.15	41.57	43.04	0.66	0.575
a*_72h_	26.13	26.19	25.07	0.32	0.294
b*_72h_	13.42	11.97	11.97	0.44	0.318
**Drip loss_24h_ (%)**	4.34^a^	3.94^b^	3.76^b^	0.09	0.018
**Drip loss_48h_ (%)**	7.30^a^	6.72^b^	6.54^b^	0.11	0.004
**Cooking loss (%)**	26.70^a^	23.57^b^	24.93^b^	0.42	0.003
**Shear force (N)**	58.26^a^	48.50^b^	50.12^b^	1.49	0.007

1 Abbreviations: Control, basal diet; 400 mg/kg, basal diet supplemented with 400 mg/kg resveratrol; 800 mg/kg, basal diet supplemented with 800 mg/kg resveratrol, L*, lightness; a*, redness; b*, yellowness.

Eighteen animals were used. Results are presented as mean, with SEM listed separately (*n* = 6 biological replicates). Distinct superscript letters within the same row denote statistically significant intergroup differences (*P *< 0.05).

### Effect of dietary RES on lipid metabolic enzyme activity of IMF in finishing steers

The adipose triglyceride lipase (ATGL) and hormone-sensitive lipase (HSL) activities in the LL muscle were lower in the 400 mg/kg RES group than in the control group (*P *< 0.05), while no significant effects were observed in the 800 mg/kg RES group (*P *> 0.05; Table 6). Conversely, acetyl-CoA carboxylase (ACC) activity in the RES groups was higher (*P *< 0.05) than in the control. No significant variations were observed in fatty acid synthase (FAS), stearoyl-CoA desaturase-1 (SCD1) and carnitine palmitoyl transferase-1 (CPT-1) enzyme activities within the LL muscle among the other groups (*P *> 0.05).

**Table 6.** Effects of dietary RES on lipid metabolic enzyme activity of intramuscular fat in finishing steers

Items^1^	Treatments			SEM	*P-*value
	Control	400 mg/kg	800 mg/kg		
**FAS, nmol/g prot**	0.29	0.27	0.32	0.01	0.099
**ACC, ng/mg prot**	0.27^b^	0.31^a^	0.32^a^	0.01	0.015
**SCD1, pg/mg prot**	34.55	33.03	32.92	0.37	0.135
**ATGL, ng/mg prot**	5.02^a^	3.92^b^	4.32^a,b^	0.17	0.015
**HSL, ng/mg prot**	0.72^a^	0.55^b^	0.76^a^	0.03	<0.001
**CPT-1, pg/mg prot**	29.31	25.68	25.65	0.87	0.145

1 Abbreviations: Control, basal diet; 400 mg/kg, basal diet supplemented with 400 mg/kg resveratrol; 800 mg/kg, basal diet supplemented with 800 mg/kg resveratrol; FAS, fatty acid synthase; ACC, acetyl-CoA carboxylase; SCD1, stearoyl-CoA desaturase-1; ATGL, adipose triglyceride lipase; HSL, hormone-sensitive lipase; CPT-1, carnitine palmitoyl transferase-1.

Eighteen animals were used. Results are presented as mean, with SEM listed separately (*n* = 6 biological replicates). Distinct superscript letters within the same row denote statistically significant intergroup differences (*P *< 0.05).

### Transcriptomics and proteomics

To clarify the mechanism by which RES promoted IMF deposition in the LL muscle of finishing steers, control and 400 mg/kg RES groups were selected for subsequent testing based on the significant differences in IMF content and lipid metabolic enzyme activity. The control group was labeled as IMF, and the 400 mg/kg RES group was labeled as IMF_RES.

#### Identification and comparison of DEGs

**Figure 2.**
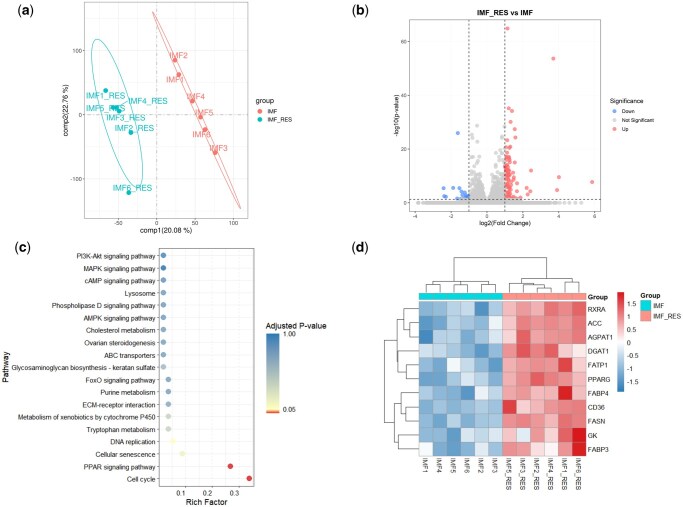
Differentially expressed genes between IMF_RES and IMF groups. (a) Principal component analysis. (b) Volcano plot of DEGs. (c) KEGG enrichment analysis of DEGs. (d) Cluster heatmap of 11 lipid metabolism-related DEGs in the PPAR signaling pathway. DEGs, differentially expressed genes; KEGG, Kyoto Encyclopedia of Genes and Genomes. IMF_RES, 400 mg/kg resveratrol group; IMF, basal diet group; RXRA, retinoid X receptor alpha; ACC, acetyl-CoA carboxylase; AGPAT1, 1-acylglycerol-3-phosphate O-acyltransferase 1; DGAT1, diacylglycerol O-acyltransferase 1; FATP1, fatty acid transport protein 1; PPARG, peroxisome proliferator-activated receptor gamma; FABP4, fatty acid-binding protein 4; CD36, cluster of differentiation 36; FASN, fatty acid synthase; GK, glycerol kinase; FABP3, fatty acid-binding protein 3.

After performing quality control of all original sequencing reads, a total of 78.5 GB of clean reads were obtained, with the average sequencing quality indicator Q30 being 97.6%. Reference genome alignment results showed that the IMF tissue transcriptome had an average genome mapping rate of 93.7%, including 91.5% unique and 2.2% multiple mapping rates (Supplementary Table S1). Principal component analysis (PCA) revealed a distinct separation between the IMF_RES and IMF groups (Figure 2a). RNA-seq analysis identified 18,842 genes, including 120 DEGs, of which 100 were upregulated and 20 were downregulated (Figure 2b). Kyoto encyclopedia of genes and genomes enrichment analysis showed significant enrichment of DEGs in the PPAR signaling pathway and cell cycle (Figure 2c). The PPAR signaling pathway is associated with lipid metabolism. Within this pathway, 11 DEGs exhibiting close associations with lipid metabolism were identified (Figure 2d): *FATP1*, cluster of differentiation 36 (*CD36*), *FABP3*, and *FABP4* were involved in fatty acid uptake and transport, while *PPARγ*, retinoid X receptor alpha (*RXRα*), *ACC*, *FASN*, glycerol kinase (*GK*), *DGAT1*, and 1-acylglycerol-3-phosphate O-acyltransferase 1 (*AGPAT1*) participated in lipid synthesis.

#### Identification and comparison of DAPs

**Figure 3.**
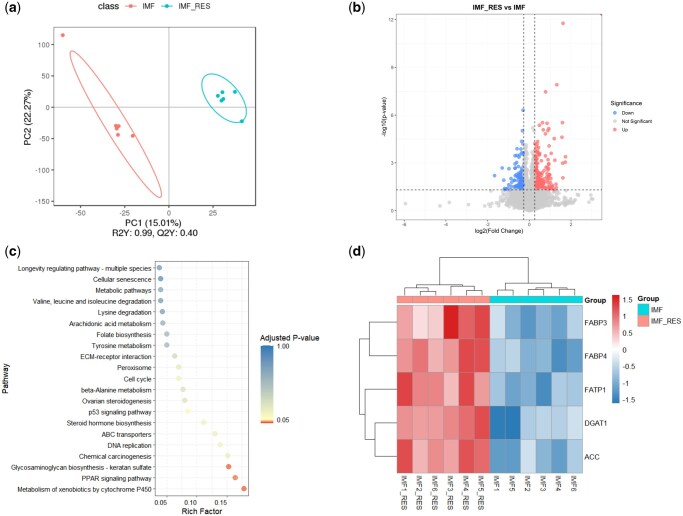
Differentially abundant proteins between IMF_RES and IMF groups. (a) Principal component analysis. (b) Volcano plot of DAPs. (c) KEGG enrichment analysis of DAPs. (d) Cluster heatmap of 5 lipid metabolism-related DAPs in the PPAR signaling pathway. DAPs, differentially abundant proteins; KEGG, Kyoto Encyclopedia of Genes and Genomes. IMF_RES, 400 mg/kg resveratrol group; IMF, basal diet group; FABP3, fatty acid-binding protein 3; FABP4, fatty acid-binding protein 4; FATP1, fatty acid transport protein 1; DGAT1, diacylglycerol O-acyltransferase 1; ACC, acetyl-CoA carboxylase.

Proteomic analysis revealed high peptide specificity and stable reproducibility of indexed retention time (iRT) values. These peptides were mapped to 9,540 proteins, and subcellular localization analysis revealed 33.6% nuclear proteins, 16.8% cytoplasmic proteins, and 6.6% mitochondrial proteins (Supplementary Figure S1). PCA revealed a distinct separation between the IMF_RES and IMF groups (Figure 3a). Further analysis identified 372 DAPs, including 249 upregulated and 123 downregulated DAPs (Figure 3b). The KEGG pathway analysis revealed that DAPs were significantly enriched in three key pathways: xenobiotics by cytochrome P450, the PPAR signaling pathway, and glycosaminoglycan biosynthesis-keratan sulfate, with the lipid metabolism-related pathway being the PPAR signaling pathway (Figure 3c). Within the PPAR pathway, 5 DAPs closely associated with lipid metabolism were identified (Figure 3d): FATP1, FABP3, and FABP4 participate in fatty acid uptake and transport, while ACC and DGAT1 are involved in lipid synthesis.

### Effects of RES on mRNA and protein expression of lipid metabolism regulatory factors in the PPAR signaling pathway

**Figure 4.**
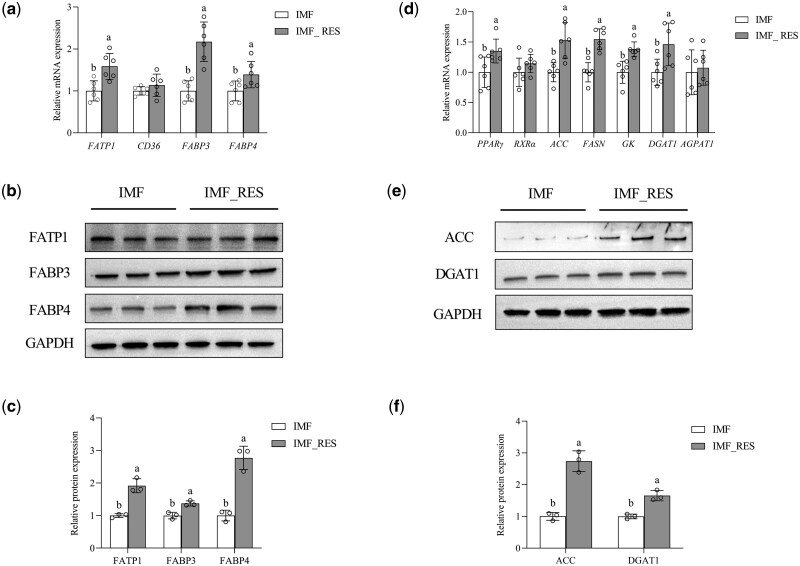
Effects of dietary resveratrol supplementation on mRNA levels (a, d) and protein abundance (b, c, e, f) of lipid metabolism-related genes in the intramuscular fat of finishing steers. Data are presented as mean ± S.E. Different lowercase letters above the bars indicate statistically significant differences between groups (*P *< 0.05). RES, resveratrol; IMF, basal diet group; IMF_RES, 400 mg/kg resveratrol group. FATP1, fatty acid transport protein 1; CD36, cluster of differentiation 36; FABP3, fatty acid-binding protein 3; FABP4, fatty acid-binding protein 4; PPARγ, peroxisome proliferator-activated receptor gamma; RXRα, retinoid X receptor alpha; ACC, acetyl-CoA carboxylase; FASN, fatty acid synthase; GK, glycerol kinase; DGAT1, diacylglycerol O-acyltransferase 1; AGPAT1, 1-acylglycerol-3-phosphate O-acyltransferase 1.

To further validate the transcriptional levels and protein expression of lipid metabolism-related factors within the PPAR signaling pathway based on the aforementioned experimental results, RT-qPCR analysis was conducted (Figure 4a and d). The results demonstrated that the mRNA expression levels of *PPARγ*, *FATP1*, *FABP3*, *FABP4*, *ACC*, *FAS*, *GK*, and *DGAT1* in the LL muscle of RES-treated finishing steers were upregulated compared with those in the control group (*P *< 0.05). In contrast, no significant intergroup differences were observed in the mRNA expression levels of other genes associated with lipid metabolism (*P *> 0.05). Western blotting analysis (Figure 4b, c, e, and f) demonstrated elevated protein expression levels of FATP1, FABP3, FABP4, ACC, and DGAT1 in the LL muscle of the RES group compared to the control group (*P *< 0.05). No significant differences were observed in the expression levels of other proteins between the experimental groups (*P *> 0.05).

## Discussion

### Effect of dietary RES on growth performance and carcass traits in finishing steers

Dietary supplementation with 400 mg/kg RES significantly increased the average daily gain and final body weight of steers. However, increasing the dosage to 800 mg/kg had no significant effect on the average daily gain. A similar dose-response relationship was observed by Shen et al. (2022), who reported that supplementing the diet of Nubian goats with 150 mg/kg RES significantly increased average daily gain, whereas increasing the dose to 300 mg/kg resulted in a nonsignificant effect. The improvement in average daily gain in the low-dose group may be associated with an increased abundance of beneficial rumen microbes involved in short-chain fatty acid production (Shen et al., 2022). However, the lack of a significant effect in the high-dose group may be attributed to the inhibition of ruminal microbial growth and fermentation function by high concentrations of phenolic compounds (Yang et al., 2010). Regarding carcass traits, dietary supplementation with 400 mg/kg RES significantly increased the live weight before slaughter and carcass weight of beef cattle while reducing backfat thickness. However, when the RES dosage was increased to 800 mg/kg, no significant differences were observed in all carcass traits indicators compared to the control group. The improvement in live weight before slaughter and carcass weights is primarily associated with improved growth performance and the reduction of backfat thickness is mainly due to the role of RES in promoting lipolysis in subcutaneous fat (Zhang et al., 2015). However, the elevated dosage of 800 mg/kg shows decreased effectiveness. These results indicate that 400 mg/kg of RES appears to be the ideal dosage. Therefore, this study establishes that dietary 400 mg/kg RES effectively improves average daily gain, carcass weight, and subcutaneous fat thickness in finishing steers, providing a scientific basis for its application in beef cattle production.

### Effect of dietary RES on meat quality and nutrient composition of LL muscle in finishing steers

Water-holding capacity is a critical quality attribute of muscle tissue. Drip and cooking losses are key evaluation parameters closely associated with both IMF, water content, and ease of water migration from the myofibrillar network (Oswell et al., 2021). Existing research indicates that dietary RES supplementation can improve meat color in the finishing pig LD muscle while simultaneously enhancing muscle water-holding capacity (Zhang et al., 2019). In the current study, the addition of RES to the diet did not affect muscle pH or color characteristics of finishing steers. However, it reduced both drip and cooking losses. Previous studies have demonstrated that RES enhances muscle antioxidant capacity and inhibits lipid oxidation (Meng et al., 2020; Cui et al., 2023). Therefore, the increased water-holding capacity of the LL muscle observed in the present study might be related to RES increasing antioxidant capacity. Shear force serves as a critical objective indicator for evaluating beef tenderness and overall eating quality (Martinez et al., 2023). Published studies have reported a range of shear force values from approximately 39.70 N to 82.1 N in the LL muscle of Angus cattle (Tayengwa et al., 2020; Bai et al., 2023; Liu et al., 2024). The shear force values obtained in the present study (48.50 N to 58.26 N) are within this range. This study found that RES significantly reduced the shear force value in the LL muscle of steers while significantly elevating IMF content. Muscle shear force is typically negatively correlated with IMF content (Shen et al., 2022). This indicates that the improvement in beef tenderness is primarily associated with RES promoting IMF deposition. In the subsequent discussion, we focused on elucidating the regulatory effects and underlying mechanisms by which RES influences IMF deposition.

### Effect of dietary RES on lipid metabolic enzyme activity of IMF in finishing steers

This study demonstrated that dietary RES supplementation in finishing steers enhanced the activity of the lipogenic enzyme ACC while suppressing the activities of lipolytic enzymes ATGL and HSL in the IMF. During lipogenesis, ACC catalyzes the conversion of acetyl-CoA to malonyl-CoA, providing an essential substrate for *de novo* synthesis, thereby significantly enhancing the rate of fatty acid synthesis (Zhang et al., 2024). ATGL and HSL, rate-limiting enzymes in triglyceride hydrolysis during lipolysis, promote fat mobilization by cleaving triglycerides into free fatty acids, thereby reducing lipid accumulation (Lasa et al., 2012). The present study demonstrated that RES modulated the activity of lipid-metabolizing enzymes, thereby enhancing lipogenesis while suppressing lipolysis, consequently increasing the IMF content.

### Effect of dietary RES on the expression of genes and proteins related to IMF deposition in finishing steers

The PPAR signaling pathway is a key lipid metabolism pathway that regulates lipid metabolism in the body through ketogenesis, lipid transport, lipogenesis, cholesterol metabolism, fatty acid transport, and fatty acid oxidation (Michalik et al., 2008). In the current study, multi-omics analysis revealed that 11 DEGs and 5 DAPs, predominantly enriched in the PPAR signaling pathway, were closely associated with fatty acid uptake, transport, and lipogenesis. These findings suggest that RES regulates lipid metabolism primarily by activating the PPAR signaling pathway, thereby promoting IMF deposition in the LL muscle of finishing steers.

Regarding fatty acid uptake and transport, integrated multi-omics and experimental validation demonstrated upregulated expression of FATP1, FABP3, and FABP4 within the IMF of LL muscle from RES-supplemented steers, in line with prior research findings (Sun et al., 2019; Wu et al., 2020). FATP1, FABP3, and FABP4 are the key factors involved in fatty acid transmembrane transport within the PPAR signaling pathway. FATP1, a crucial transmembrane transporter that mediates long-chain fatty acid uptake, significantly enhances fatty acid absorption in muscle tissues when its expression is elevated (Zhong et al., 2024). FABP3 and FABP4 mainly bind to intracellular free fatty acids and promote their transport to lipid droplets (Wang et al., 2023). Therefore, we hypothesized that RES promotes IMF deposition, in part, by enhancing fatty acid uptake and transport.

For fat synthesis, transcriptomics and RT-qPCR validation showed that the levels of *PPARγ*, *ACC*, *FAS*, *GK*, and *DGAT1* were significantly increased in the RES group. Proteomics and western blotting validation revealed significantly elevated expression of ACC and DGAT1. PPARγ is a key regulator of IMF deposition. It forms heterodimers with RXRα that recognize and bind to specific DNA sequences, thereby activating downstream adipogenic gene expression (Lefebvre et al., 2010). FAS is a key rate-limiting enzyme in the long-chain fatty acid synthesis pathway that utilizes malonyl-CoA generated by ACC catalysis for fatty acid synthesis (Witkowski et al., 2007; Huh & Saltiel, 2021). GK catalyzes the generation of glycerol-3-phosphate from glycerol, providing a basic backbone for triglyceride synthesis (Miao et al., 2019). DGAT1 catalyzes the formation of triglycerides from fatty acids and glycerol, thereby promoting lipid droplet formation (Guo et al., 2023). Thus, the mechanism of action of RES in promoting IMF deposition is, in part, through the modulation of the expression of PPARγ and its downstream lipid synthesis factors.

## Conclusion

The addition of 400 mg/kg RES to the diet of finishing steers improved growth performance and carcass traits, while enhancing the muscle quality of the LL muscle and significantly increasing the IMF content. Multi-omics analysis and validation results demonstrated that the PPAR signaling pathway is a key pathway through which RES promotes IMF deposition, primarily by enhancing fatty acid uptake and transport, as well as fat synthesis. This study provides a theoretical basis for promoting IMF deposition of finishing steers and improving beef quality. It should be noted that this study was limited by the relatively small sample size. Therefore, further studies with larger sample sizes are warranted to validate these findings.

## Supplementary Material

skaf410_Supplementary_Data

## Abbreviations

ACC: acetyl-CoA carboxylase
AGPAT1: 1-acylglycerol-3-phosphate O-acyltransferase 1
ATGL: adipose triglyceride lipase
CD36: cluster of differentiation 36
CPT-1: carnitine palmitoyl transferase-1
DAPs: differentially abundant proteins
DEGs: differentially expressed genes
DGAT1: diacylglycerol O-acyltransferase 1
FABP3: fatty acid-binding protein 3
FABP4: fatty acid-binding protein 4
FASN: fatty acid synthase
FATP1: fatty acid transport protein 1
GK: glycerol kinase
HSL: hormone-sensitive lipase
IMF: intramuscular fat
KEGG: kyoto encyclopedia of genes and genomes
LL: *Longissimus lumborum*
PBS: phosphate-buffered saline
PCA: principal component analysis
PPAR: peroxisome proliferator-activated receptor
PPARγ: peroxisome proliferator-activated receptor gamma
RES: resveratrol
RT-qPCR: real-time quantitative PCR
RXRα: retinoid x receptor alpha
SCD1: stearoyl-CoA desaturase-1
SIRT1: sirtuin 1
