# Supplementary material for: Effects of resveratrol on growth performance, meat quality, and intramuscular fat deposition in finishing steers
Source: J Anim Sci. 2025 Nov 25;103:skaf410. doi: 10.1093/jas/skaf410 (PMC12711393; doi:10.1093/jas/skaf410)
Supplement: skaf410_Supplementary_Data [file skaf410_supplementary_data.docx]

Table S1 Quality Control of Transcriptomics Data

| sample | clean_reads | clean_bases | Q30 | GC_pct | total_map | unique_map | multi_map | positive_map | negative_map | splice_map | unsplice_map |
| --- | --- | --- | --- | --- | --- | --- | --- | --- | --- | --- | --- |
| IMF1 | 46273414 | 6.94G | 97.43 | 52.26 | 42856113(92.61%) | 41849863(90.44%) | 1006250(2.17%) | 20898280(45.16%) | 20951583(45.28%) | 19986434(43.19%) | 21863429(47.25%) |
| IMF2 | 40568494 | 6.09G | 97.23 | 52.61 | 37978154(93.61%) | 37096277(91.44%) | 881877(2.17%) | 18531324(45.68%) | 18564953(45.76%) | 18268142(45.03%) | 18828135(46.41%) |
| IMF3 | 45907588 | 6.89G | 97.51 | 51.34 | 42972828(93.61%) | 41965572(91.41%) | 1007256(2.19%) | 20962206(45.66%) | 21003366(45.75%) | 20261466(44.14%) | 21704106(47.28%) |
| IMF4 | 44839792 | 6.73G | 97.66 | 52.43 | 41631505(92.85%) | 40632506(90.62%) | 998999(2.23%) | 20293067(45.26%) | 20339439(45.36%) | 20143300(44.92%) | 20489206(45.69%) |
| IMF5 | 46620162 | 6.99G | 97.7 | 51.89 | 43703079(93.74%) | 42663368(91.51%) | 1039711(2.23%) | 21311107(45.71%) | 21352261(45.8%) | 20929106(44.89%) | 21734262(46.62%) |
| IMF6 | 48565116 | 7.28G | 97.62 | 52.05 | 45399553(93.48%) | 44334997(91.29%) | 1064556(2.19%) | 22140234(45.59%) | 22194763(45.7%) | 21776707(44.84%) | 22558290(46.45%) |
| IMF1_RES | 46282086 | 6.94G | 97.65 | 51.98 | 43495221(93.98%) | 42508881(91.85%) | 986340(2.13%) | 21233493(45.88%) | 21275388(45.97%) | 20007259(43.23%) | 22501622(48.62%) |
| IMF2_RES | 46249262 | 6.94G | 97.61 | 52.41 | 43385761(93.81%) | 42399414(91.68%) | 986347(2.13%) | 21179152(45.79%) | 21220262(45.88%) | 20596991(44.53%) | 21802423(47.14%) |
| IMF3_RES | 48071556 | 7.21G | 97.54 | 52.67 | 45229304(94.09%) | 44185601(91.92%) | 1043703(2.17%) | 22076028(45.92%) | 22109573(45.99%) | 21641149(45.02%) | 22544452(46.9%) |
| IMF4_RES | 51016320 | 7.65G | 97.63 | 52.56 | 48112951(94.31%) | 46991270(92.11%) | 1121681(2.2%) | 23475543(46.02%) | 23515727(46.09%) | 22799393(44.69%) | 24191877(47.42%) |
| IMF5_RES | 47102726 | 7.07G | 97.57 | 52.55 | 44333948(94.12%) | 43307636(91.94%) | 1026312(2.18%) | 21632716(45.93%) | 21674920(46.02%) | 21051285(44.69%) | 22256351(47.25%) |
| IMF6_RES | 47880890 | 7.18G | 97.77 | 49.24 | 44856617(93.68%) | 43891843(91.67%) | 964774(2.01%) | 21928603(45.8%) | 21963240(45.87%) | 19058508(39.8%) | 24833335(51.86%) |

Sample, Sample identifier; Clean_reads, Number of high-quality reads after raw data filtering; Clean_bases, Total bases in clean reads; Q30, Percentage of bases with Phred quality score >30; GC_pct, GC content (%) in clean reads. total_map: The number of reads aligned to the genome and their percentage; unique_map: The number of reads uniquely mapped to a single location in the reference genome and their percentage (used for subsequent quantitative data analysis of reads); multi_map: The number of reads mapped to multiple locations in the reference genome and their percent.


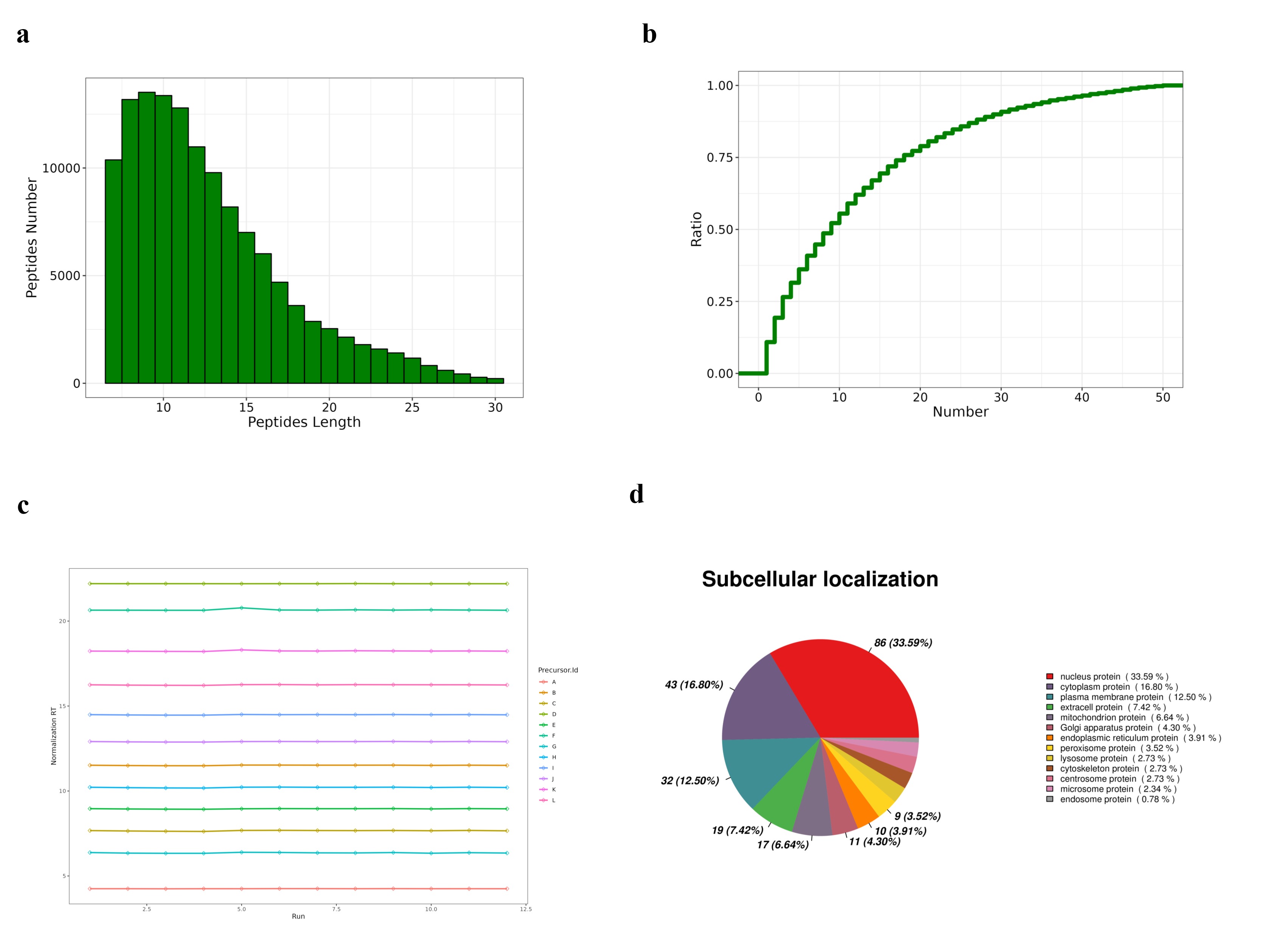


**Fig. S1.** Quality control of proteomics data (a, b, c, d). (a) displays the distribution of peptide length ranges, where the x-axis represents the number of amino acid residues in peptides, and the y-axis indicates the count of peptides at each specific length. (b) illustrates the distribution of unique peptides identified in proteins. The x-axis corresponds to the number of unique peptides, while the y-axis shows the cumulative percentage of proteins containing unique peptides relative to the total protein count as the number of unique peptides increases. (c) presents the iRT (indexed Retention Time) values of internal standard-calibrated peptides. The x-axis indicates sample identifiers, and the y-axis represents the calibrated iRT values of the internal standard peptides. (d) subcellar localization.Biological cells exhibit a highly compartmentalized architecture, wherein intracellular components are spatially segregated into distinct membrane-bound organelles or functional domains based on their topological organization and specialized physiological roles, including but not limited to the nucleus, Golgi apparatus, endoplasmic reticulum, mitochondria, cytosol, and plasma membrane.
